# DEVILS: a tool for the visualization of large datasets with a high dynamic range

**DOI:** 10.12688/f1000research.25447.2

**Published:** 2021-03-22

**Authors:** Romain Guiet, Olivier Burri, Nicolas Chiaruttini, Olivier Hagens, Arne Seitz

**Affiliations:** 1 BioImaging & Optics platform (BIOP), Faculty of Life Sciences (SV), Ecole Polytechnique Fédérale de Lausanne (EPFL), Lausanne, Switzerland; 2 Laboratory of Neural Microcircuitry, Ecole Polytechnique Fédérale de Lausanne, Lausanne, Switzerland

**Keywords:** Large datasets, ImageJ/Fiji, Image Processing, BigDataViewer, Light-sheet fluorescence microscopy

## Abstract

The number of grey values that can be displayed on monitors and be processed by the human eye is smaller than the dynamic range of image-based sensors. This makes the visualization of such data a challenge, especially with specimens where small dim structures are equally important as large bright ones, or whenever variations in intensity, such as non-homogeneous staining efficiencies or light depth penetration, becomes an issue.

While simple intensity display mappings are easily possible, these fail to provide a one-shot observation that can display objects of varying intensities. In order to facilitate the visualization-based analysis of large volumetric datasets, we developed an easy-to-use ImageJ plugin enabling the compressed display of features within several magnitudes of intensities. The Display Enhancement for Visual Inspection of Large Stacks plugin (DEVILS) homogenizes the intensities by using a combination of local and global pixel operations to allow for high and low intensities to be visible simultaneously to the human eye.

The plugin is based on a single, intuitively understandable parameter, features a preview mode, and uses parallelization to process multiple image planes. As output, the plugin is capable of producing a BigDataViewer-compatible dataset for fast visualization.

We demonstrate the utility of the plugin for large volumetric image data.

## Introduction

The display of data from a light microscope is challenging as the modulation transfer function, which dictates the contrast, is size dependent. Images of small objects have intrinsically a lower contrast than larger ones. In particular, objects with a size close to the resolution limit of the imaging system are hardly contrasted, especially when imaged simultaneously with larger objects (Figure S1, see *Extended data* ( Guiet *et al.*, 2020a)). This intrinsic drawback is even reinforced by the fact that the labelling efficiency might not be uniform for small and large structures. Moreover, optical aberrations, in particular spherical ones, further degrade the signal in a non-linear and sample-dependent manner due to refractive index mismatches. The visual inspection of such image data is therefore challenging. This is often the case for large volumetric datasets as typically provided by light-sheet fluorescence microscopy (LSFM).

In LSFM, detection and image acquisition of the images is camera based, delivering images with a bit depth of 12 bits and beyond. The resulting image stacks can therefore exhibit a dynamic range that is larger than the grey values that can be distinguished by the human visual system or that can be displayed on monitors and screens.

Fiji is one of the mostly used open source software for biological image data ( Schindelin *et al.*, 2012). However, it struggles with the display and rapid inspection of large volumetric datasets with a high dynamic range. We therefore propose a Fiji plugin that facilitates on-screen display of structures with intensities differing by several orders of magnitude called Display Enhancement for Visual Inspection of Large Stacks (DEVILS).

DEVILS performs local tone-mapping of fluorescence microscopy images, which has been commonly applied in every day world ( Salih *et al.*, 2012) and medical image processing ( Park & Montag, 2007). To achieve this in fluorescence images from biological samples, DEVILS uses a combination of three image processing routines. The original image is divided by a processed copy of itself (i.e. convolved with a Gaussian kernel) followed by a nonlinear intensity modification (square root operation) and a local rolling-ball background subtraction. Together they are implemented as a Fiji plugin. To deal with the size of the datasets, in the range of hundreds of gigabytes, the plugin can work on multiple planes of a virtual stack in parallel threads.

We compare the result of the operations with several well-known and established methods to adjust the display of an image and furthermore analyse the effect of the DEVILS algorithm by comparing objects varying in intensity and size, as well as the different noise and background levels in the image. Details of the operations and its implementation as a user-friendly ImageJ plugin are discussed afterwards.

## Methods

### Microscopy

**Figure 1.**
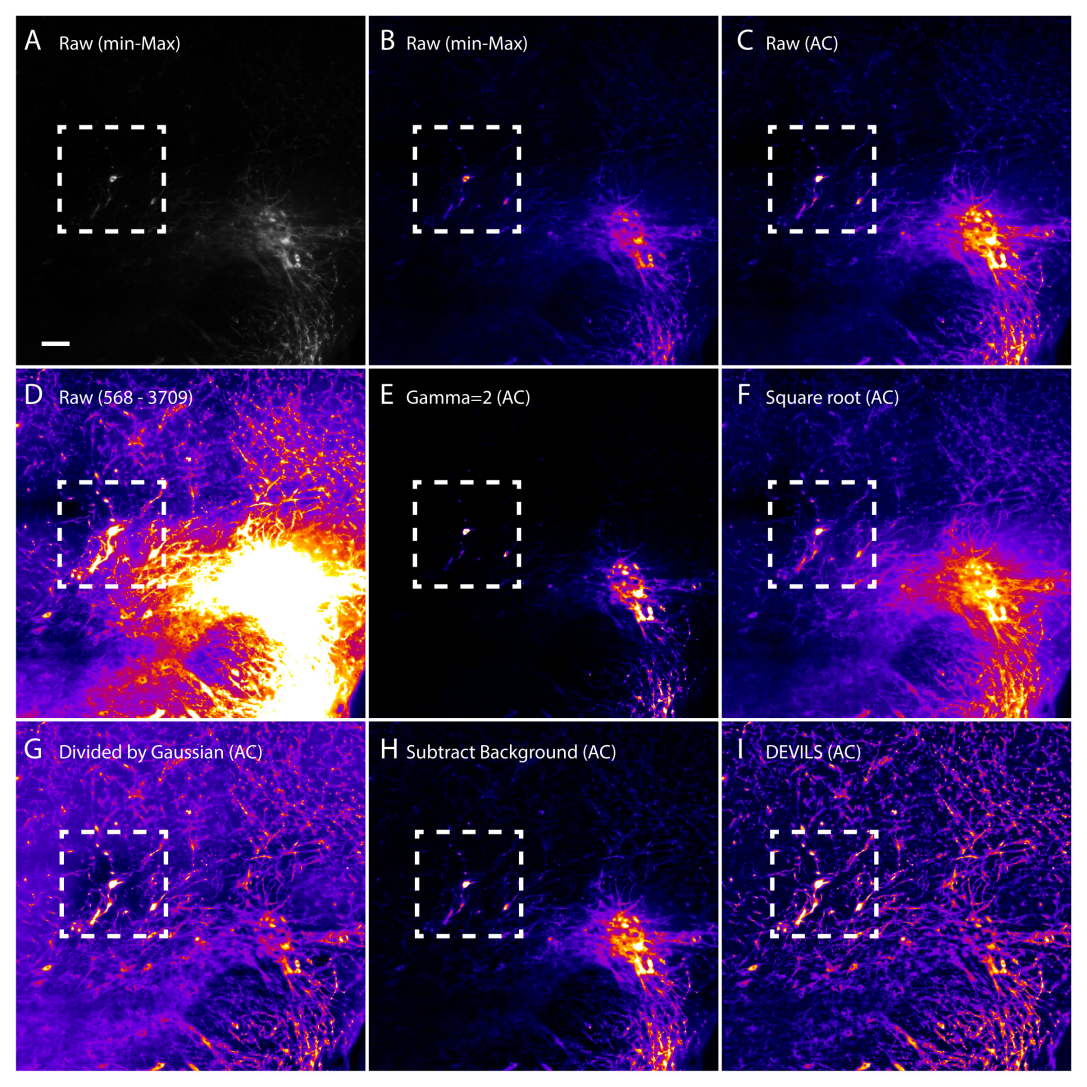
Comparison of different image display methods. An optical slice of a chemically cleared mouse midbrain was imaged with light-sheet fluorescence microscopy (LSFM) and different image processing operations are compared. Images are displayed using the Fire look-up table (LUT) except for A which is displayed in grey-scale. Apart from panels **A**, **B** and **D**, the auto contrast (AC) adjustment of Fiji was set to 0.35. ( **A**) Unprocessed image displayed using a grey LUT with the minimum (min) and maximum (max) display settings set to the pixel histogram values (568 – 55656). Scale bar: 100 µm. ( **B**) Unprocessed image displayed using the Fire LUT with the same display settings as in **A**. ( **C**) Unprocessed image displayed using AC adjustment. ( **D**) Unprocessed image displayed with min-max set to 568 – 3709 (3709 equals 1/15 ^th^ of the maximal intensity). ( **E**) Original image displayed using Gamma adjustment (=2). ( **F**) Original image after applying the Square Root operation. ( **G**) Original image after a division by a spatially filtered version of the image (Gaussian blur, =50). ( **H**) Original image after applying the ‘Subtract Background’ method from Fiji/ (radius = 25 px). ( **I**) Original image after applying the DEVILS plugin with *p* = 25.

***Cleared right murine midbrain, using active clarity protocol ( Lee *et al.*, 2016) ( Figure 1 and Figure 3).*** Multichannel stacks were acquired as tiles with 15% overlap on a Zeiss Lightsheet Z1 using a 20×/1.0 clearing objective. Each channel was acquired sequentially for each slice using single-sided illumination and a lightsheet thickness of 5.5 μm at the center and an optic zoom of 0.75×. The channels were acquired in the following order: tdTomato, endogenous staining of dopaminergic neurons (Ex: 561 nm, Em: BP 575 – 615 nm); Alexa Fluor 488, immunostaining of TH (Ex: 488 nm, Em: BP 525 – 545 nm) and DAPI, nuclear counterstain (Ex: 405 nm, Em: 420 – 470 nm). Original acquisitions yielded voxel sizes of 0.29 μm in XY and 1.5 μm in Z. The resulting tiles were fused and downscaled to an isotropic resolution of 1.2 μm using BigStitcher ( Hörl *et al.*, 2019).

***Cleared whole mouse (Parvalbumin-Cre C57BL/6) brain using CLARITY1 protocol ( Figures 5A and 5B).*** Single channel stack acquisition on a mesoSPIM system ( Voigt *et al.*, 2019) (Wyss Center Advanced Light-sheet Imaging Center, Geneva, Switzerland) using a 42 Olympus MVX-10 zoom macroscope with a 1× objective (Olympus MVPLAPO 1×), for a final magnification of 0.8× for Figure 5A and 2.0× for Figure 5B. Stacks were acquired in single-sided illumination. The acquired channel represents the expression of php.eB AAV ( Chan *et al.*, 2017) through endogenous expression of TdTomato (Ex: 561 nm, EM: LP 561 nm, BrightLine HC, AHF). Final voxel sizes are 8.23 μm in XY and 5 μm in Z for Figure 5A, and 3.26 μm in XY, and 3 μm in Z for Figure 5B. pAAV-FLEX-tdTomato was a gift from Edward Boyden (Addgene plasmid # 28306 ; http://n2t.net/addgene:28306 ; RRID:Addgene_28306)

***Drosophila larva brain ( Figure 5C).*** The sample was dissected in PBS and suspended in a 2 mm diameter capillary with 1% agarose and imaged on a Zeiss Lightsheet Z1 using a 20×/1.0 water objective. A single channel with dual-sided illumination was acquired in GFP over a Z-stack. This acquisition was repeated over four angles (multiview acquisition) at 90° intervals. The acquired channel represents endogenous expression of nuclear GFP on a subset of motoneurons (Ex: 488 nm Em: BP 525 – 545 nm). Original acquisitions yielded voxel sizes of 0.46 μm in XY and 1.16 μm in Z. The resulting multiview acquisition was registered and fused to an isotropic voxel size of 0.46 μm using BigStitcher ( Hörl *et al.*, 2019).

### Common display methods and comparison with DEVILS

One of the benefits of LSFM is that large specimens can be imaged at subcellular resolution. However, the modulation contrast obtained with diffraction limited light microscopy scales with the size of the imaged objects. An object with a size equal to the diffraction limit of the imaging system is half as bright compared to objects with two times the size of the diffraction limit. The intensity of objects with sizes half the diffraction limit is a mere 10% compared to the larger objects mentioned earlier ( Williams & Becklund, 2002). Adding variation of biomarker expression and staining efficiency to the equation makes it clear that images of specimens with both large and small objects will contain areas whose intensity differs by several orders of magnitude. Brain tissue constitutes a prominent example (see Figure 1). Regions with clusters of cells with high pixel intensities and rather dim individual cells typically make displaying the image even more challenging. In the following section, we will compare common display modifications and outline the DEVILS plugin.

In Figure 1 one optical plane of a chemically cleared part of a mouse brain is displayed, acquired with a LSFM. Meaningful inspection of the dataset requires use of the entire dynamic range of the camera throughout the acquisition and its processing for inspection. Figure 1A displays the image data using a grey-scale lookup table (LUT), using the minimum and maximum pixel value as the upper and the lower limit for the display mapping function. This procedure is not giving satisfactory results as only the bright structures in the lower right corner are visible.

Better visualisation of image data is obtained with a coloured LUT as the human eye can distinguish more colours than grey scales. This standard approach exploits the 24 bits of information (RGB colour) to render a wide range of intensities of a single channel visible ( Silva *et al.*, 2011). Figure 1B shows the application of the “Fire” LUT from Fiji, using the minimum and the maximum pixel value as the upper and the lower limit for the display mapping function. Comparison with Figure 1A reveals that faint structures can be better recognized in the coloured image. However, the improvement is not sufficient to display all image data. Figure 1C and 1D illustrate that more structures become visible when adjusting the linear display function. These linear display adjustments combined with coloured LUTs are still not sufficient to display all of the image information: structures that are not visible in the top right corner of the image in Figure 1B and 1C become visible in Figure 1D when the maximum of the display function is lowered. However, clipping artefacts are visible at the same time for the brightest structures in the middle of the image.

Another well-known approach to improve visualization is the application of non-linear display functions. The most common one is gamma correction, which modifies the display exponentially using a value ɣ as the exponent of the original pixel intensity. Using ɣ >1 facilitates the recognition of bright structures. Thus, it can be used to suppress a homogeneous background in the sample (e.g. out of focus light, autofluorescence or unspecific antibody staining). However, it is of note that dim structures disappear ( Figure 1E). To help visualise dim structures, ɣ <1 is better suited. For example, using ɣ = 0.5, mathematically identical to calculating the square root of the image, helps to identify faint objects in the top right part of the image ( Figure 1F). Note that fainter objects are easier to identify than in the aforementioned images using linear display settings ( Figure 1A – D).

A suitable method to correct unwanted background intensity values is to divide the original image by a blurred version of itself. The resulting image of such an operation is shown in Figure 1G. The operation acts as a high-pass filter. It requires careful selection of the width of the gaussian filter and can lead to artefacts on the edge of objects, as well as an increase of the background signal and of the noise in the faintest areas of the original image. Using a so-called ‘Subtract Background’ method (aka rolling ball method) one can avoid such undesired perception effects ( Figure 1H) but this does not help much with reducing the range between the low and high intensity values.

Local contrast enhancement as implemented in the CLAHE plugin of Fiji/ImageJ turned out to be non-trivial in finding the right parameters (see Supplementary Figure S2.). Therefore, we propose here a simple workflow that homogenizes the intensities and removes background so that high and low intensities are visible simultaneously for the human eye. It requires one parameter *p*, the pixel size of the object of interest. The obtained result is the image Figure 1I that allows the observer to visualize low and high intensity objects without further adjustments.

###  DEVILS implementation

**Figure 2.**
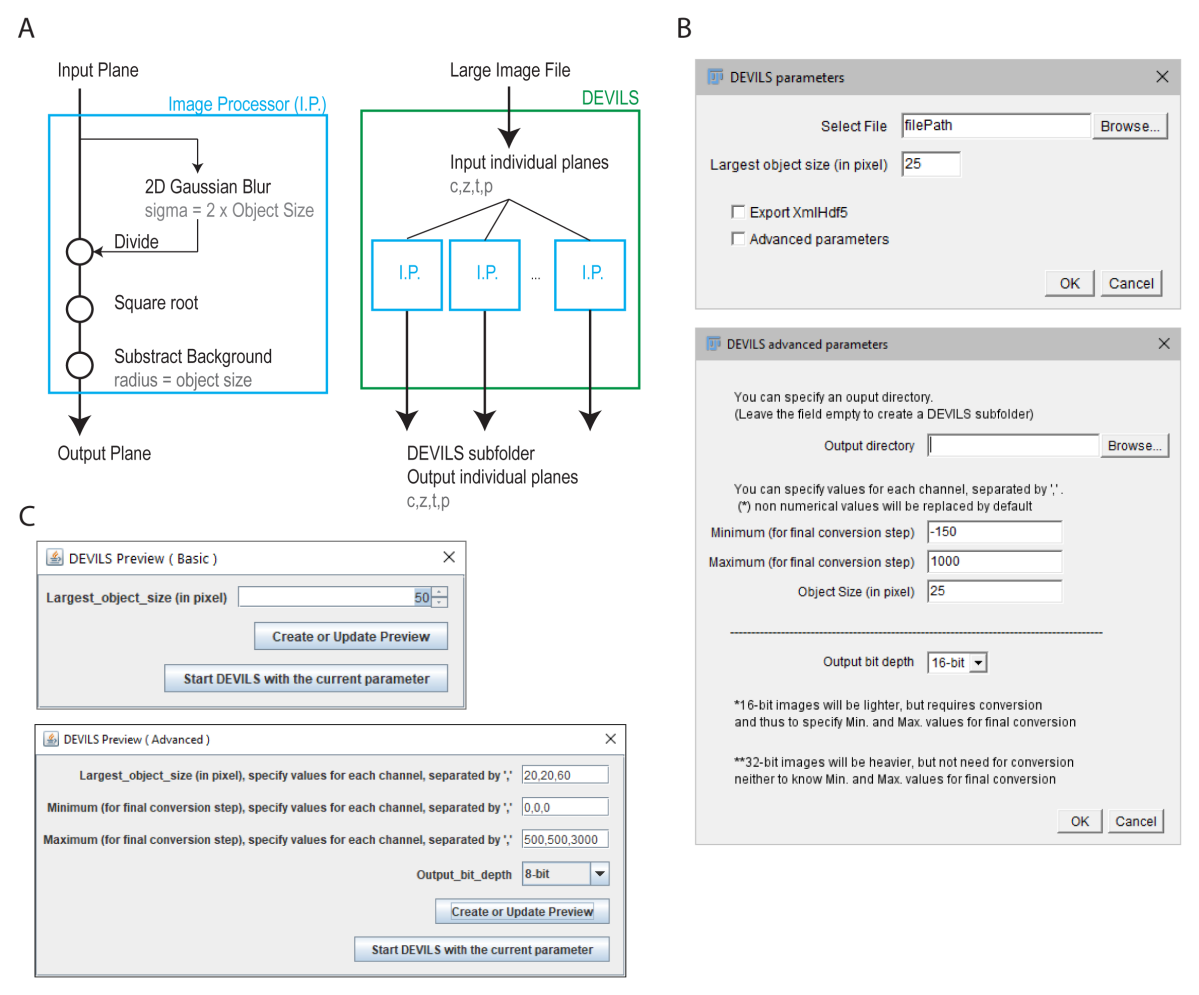
Implementation of DEVILS in ImageJ/Fiji. ( **A**) Schematic workflow of the DEVILS ImageProcessor (IP) operation and its parallelization in order to handle large image stacks. ( **B**) Graphical user interface of the plugin. In the standard mode, only the image location and the parameter *p* is required from the user. The advanced mode enables the user to define the output directory, define an object size per channel, minimum and maximum values for conversion (necessary with 8-bit and 16-bit output) and the output bit depth (8-, 16- or 32-bit). ( **C**) Graphical user interface of the preview plugin.

Our current implementation of the aforementioned operations is a Fiji plugin that uses a simple image processor and parallelizes its processing on the available cores of the machine ( Figure 2A). This enables the processing of large image stacks in a reasonable amount of time: it takes seven minutes to process a 12 GB stack on a workstation typically used for image processing. The plugin reads the selected file as a virtual stack and accesses each individual plane for processing. Only one parameter *p* is needed. It shall correlate with the size of the largest object in the image, in pixels. The plugin performs a division by a Gaussian blurred version of the image (=2p), calculates the square root and subtracts the background using the rolling ball method as implemented in Fiji, with a radius equal to *p*. The output images are then exported as individual tif files. A virtual stack reconstituted from the individual tif files can be opened with a ‘Open DEVILS Folder’ command. Furthermore, images can also be exported in the Hdf5 format, which allows their inspection with the BigDataViewer ( Pietzsch *et al.*, 2015).

All parameters are entered via a graphical user interface (GUI) as shown in Figure 2B and are recordable as a macro. The advanced parameter options allow the user to specify the output folder, change the output bit-depth of the images (8-, 16- or 32-bit) and specify the minimum and maximum ranges for conversion to an 8-bit or 16-bit image. Such a conversion decreases the size of the output data but requires careful selection of the minimum and maximum values to avoid data clipping artefacts. In the basic mode, a minimum and maximum of -100 and 10000, respectively, are set and the image bit depth is fixed at 16-bit. For rapid testing of the DEVILS operation, a preview mode is available ( Figure 2C). It processes a single image and can be used for parameter optimization e.g. to find suitable values for the image conversion.

### Operation

A machine with 8GB of RAM or above will be able to run DEVILS, provided it has enough RAM to contain a single XY plane of your data at any time. DEVILS requires Fiji to run, and can be installed by checking the PTBIOP update site under Help > Update > Manage Update Sites ( https://imagej.net/Update_Sites).

### Synthetic images

**Figure 3.**
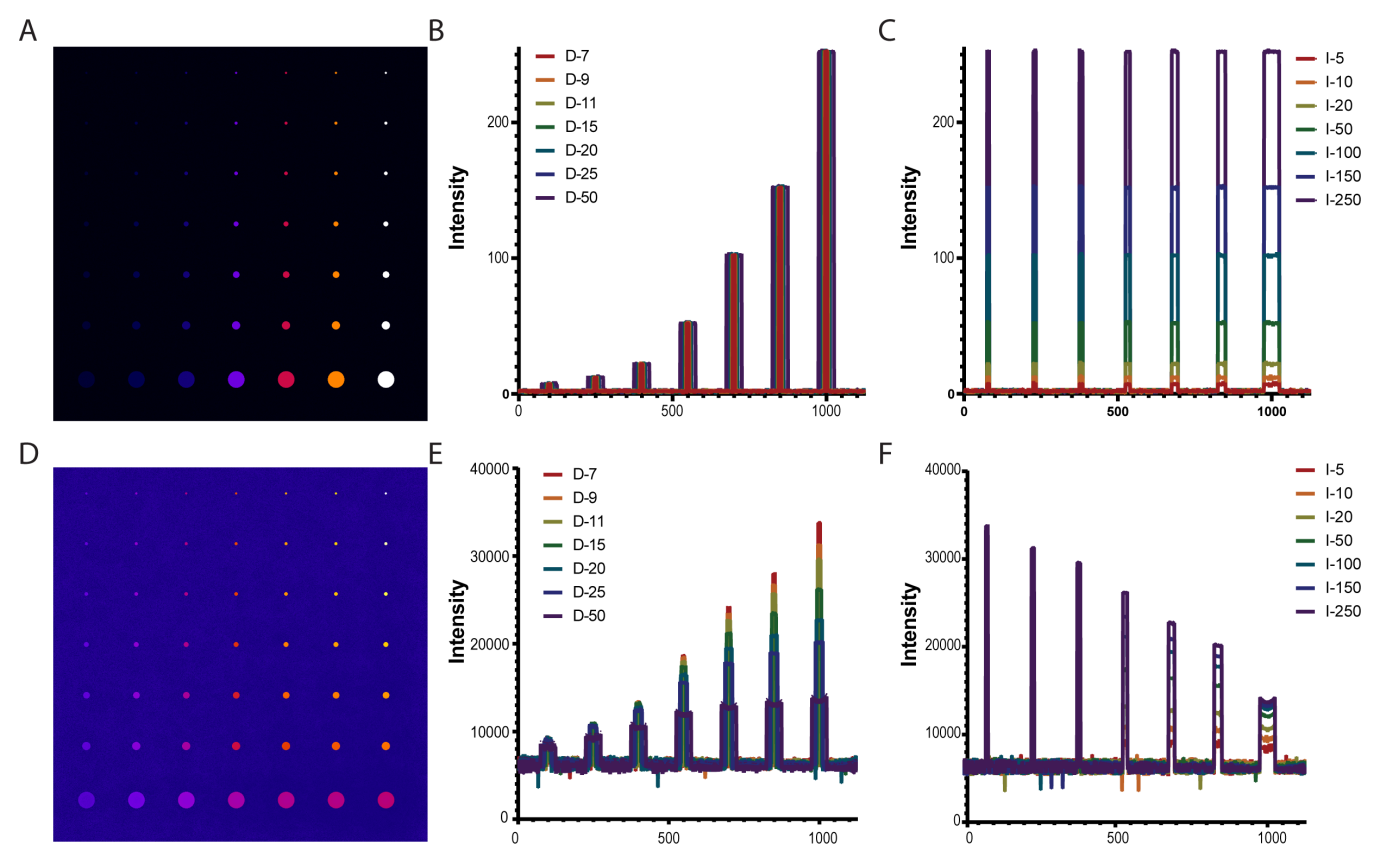
Synthetic images to illustrate the effect of DEVILS. ( **A**) Synthetic image of disks with increasing pixel intensity values from left to right (5, 10, 20, 50, 100, 150, 250) and of increasing diameter from top to bottom (7 px, 9 px, 11 px, 15 px, 20 px, 25 px, 50 px). Display settings are set to the minimum and maximum intensity values of the image and the “Fire” LUT is used for display. ( **B**) Vertical line profiles over disks shown in **A**. The different diameters are labelled with different colours. ( **C**) Horizontal line profiles over disks shown in **A**. the different intensities are labelled with different colours. ( **D**) Image **A** processed with the DEVILS plugin ( *p* = 25). ( **E**) Vertical line profiles over disks shown in **D**. The different diameters are labelled with different colours. ( **F**) Horizontal line profiles over disks shown in **D**. the different intensities are labelled with different colours.

To better understand the effects of the DEVILS plugin on image data, we applied it to a synthetic image containing disks with increasing intensities (5 – 250) and with increasing diameters (7 – 50 pixels) ( Figure 3A). A background of 2 intensity units was added and the entire signal was subjected to Gaussian noise with a standard deviation of 0.5. Despite the use of the “Fire” LUT, it is impossible to observe the smallest and faintest spots in the original 8-bit image. The intensity profiles for each row of disks with varying diameter are plotted in Figure 3B using different colours (smaller diameter in red, larger diameter in violet). The profiles are similar with intensities increasing from left to right independent of their respective diameters. Similar results are obtained for vertical intensity profiles ( Figure 3C). The intensity of the disks is independent of its size.

The effect of the DEVILS plugin is visible in Figure 3D, with the smaller and fainter spots now being visible (top left corner of the image) and the intensity of the larger and brighter spots being compressed. The line profiles in Figure 3E – F reveal that the DEVILS output is scaling with the size and the intensity of the input disk. It becomes obvious that larger disks and brighter disks become dimmer after DEVILS processing. The intensity amplification factor (see Figure S5, *Extended data* ( Guiet *et al.*, 2020a)) is larger for low intensity objects of small size. In the example shown here the ratio of input to output object intensity is around two for the dimmest and smallest object ( *r* = 7, *I* = 5). It drops to 1.6 for the largest object with the same intensity. It drops more markedly with increasing intensity. It is around one for *I* = 20 and drops to 0.25 for the smallest disk with the maximum intensity ( *r* = 7, *I* = 250). The intensity of the largest object ( *r* = 50) is damped by a factor of 0.07. In summary: small, dim objects are becoming brighter, the pixel intensity of large, bright objects is reduced. The maximal amplification difference between the smallest dimmest object and the largest brightest object is 30 in the example shown. This enables the researcher to inspect these objects in one single image. However, it must be stressed that the intensity of the signal can by no means be correlated to the protein, antigen or antibody concentration after the DEVILS plugin has been applied.

Furthermore, the parameter *p* needs to be carefully chosen. In case it is smaller than the largest objects, artefacts in these objects can be observed. The line profile after DEVILS treatment ( *p* = 25) of the disks with a diameter of 50 pixels indicate that the intensity inside the disk is lower than at the edges. This effect can be avoided by increasing the parameter *p* to 50 or above.

## Use cases

### Multichannel images

**Figure 4.**
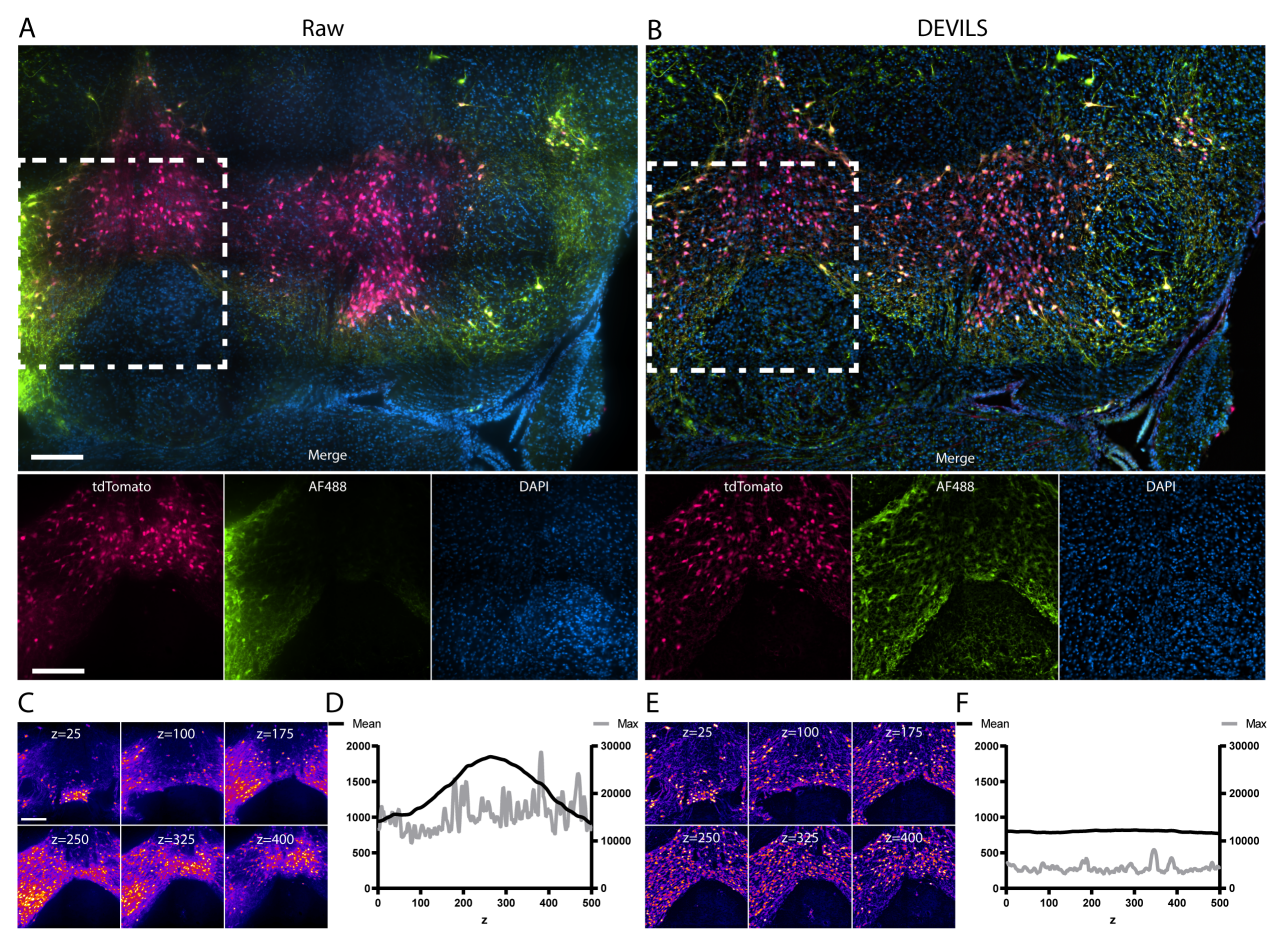
DEVILS applied to a multichannel image. A part of a cleared mouse midbrain was imaged using light-sheet fluorescence microscopy (LSFM). ( **A**) Raw and merged display of the three individual channels of a single optical slice. The DAPI nuclear counterstain is false coloured in azure, the α-TH staining is shown in chartreuse (AF488) and the dopaminergic neurons in bright pink (tdTomato). The image is assembled from 24 individual tile images. The dashed square indicates a region used for cropping. Crops for each of the individual channels are shown below the main panel. Scale bars: 250 µm. ( **B**) Image **A** after DEVILS processing ( *p* = 25). ( **C**) Cropped region from **A** showing the dopaminergic neurons at different Z-positions in the image stack, using the “Fire” look-up table (LUT) and auto contrast adjustment set to 0.35 on slice 280. Scale bar: 250 µm. ( **D**) Mean (black) and maximum (Max, grey) intensities (left and right Y-axes, resp.) of the acquisition channel as shown in panel **C** per Z-plane (X-axis). ( **E**) Cropped region from **B** showing the dopaminergic neurons at different Z-positions in the image stack, using the Fire LUT and auto contrast adjustment set to 0.35. ( **F**) Mean (black) and maximum (Max, grey) intensities (left and right Y-axes, resp.) of the acquisition channel as shown in panel **E** per Z-plane (X-axis).

**Figure 5.**
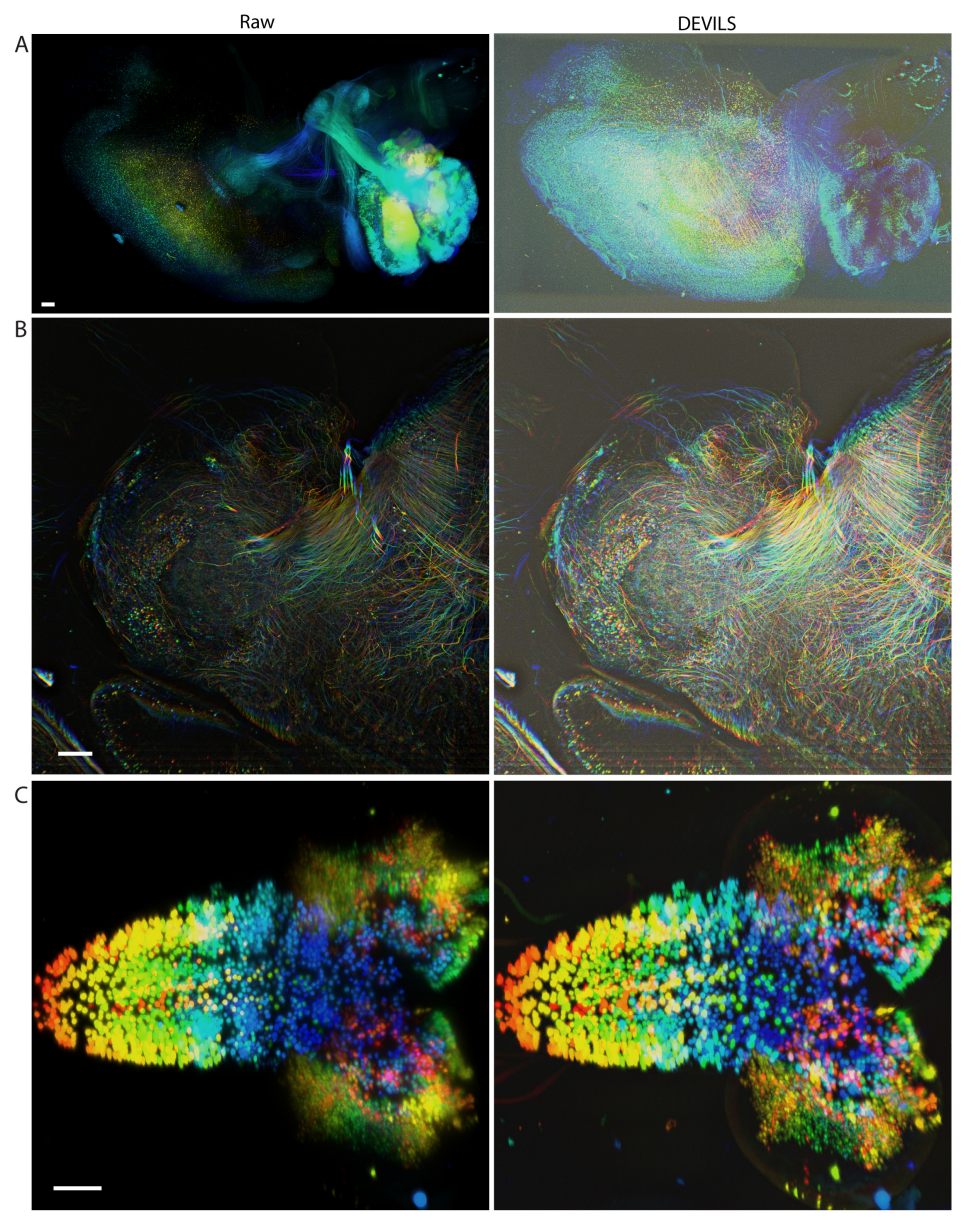
Example images of DEVILS processing. ( **A**) Cleared mouse brain acquired with a mesoSPIM\0.8X. Comparison of raw and DEVILS processed images of a Z-color-coded projection using the “Physics” look-up table (LUT). The auto contrast was set on slice 600. Scale bar: 500 µm. ( **B**) Cleared mouse brain acquired with a mesoSPIM\2.0X. Comparison of raw and DEVILS processed images of a Z-color-coded projection using the “Physics” LUT. The auto contrast was set on slice 600. Scale bar: 500 µm. ( **C**) *Drosophila* brain acquired with light-sheet fluorescence microscopy (LSFM). Comparison of raw and DEVILS processed images of a Z-color-coded projection using the Physics LUT. The auto contrast was set on slice 380. Scale bar: 50 µm.

In Figure 4 the DEVILS plugin was used in order to facilitate the inspection of a 3D multichannel dataset. Dopaminergic neurons in the mouse midbrain expressing the fluorescent protein tdTomato were immunostained with an α-tyrosine hydroxylase (TH) antibody, visualized with an Alexa Fluor 488 secondary antibody (AF488). DAPI was used as a nuclear counterstain. The plugin was applied to each channel separately. The overlay of the raw images and the outcome of the DEVILS operation are shown in the main panels of Figure 4A and 4B, respectively. Several observations can be made when comparing cropped regions from raw and processed images (dashed square in Figure 4A – B). First, DEVILS processing reveals a lot of hidden detail; this is visible in all channels, but most marked in the AF488 channel. Second, the intensity drop of individual acquisition tiles towards their edges is reduced after DEVILS processing. Third, a final advantage can be seen in the intensity profile plots along the Z-axis of the image stack: DEVILS processing significantly flattens the bell-shaped curve of the mean intensity per plane ( Figure 4D and 4F). This enables a more rapid inspection of the 3D-dataset as no intensity adjustments need to be made when moving between planes. In fact, the entire dataset can now be visualized with the same display settings. The utility of DEVILS is furthermore demonstrated in Figure 5 where it was applied to datasets acquired from different species and different microscope setups.

## Conclusion

We presented the DEVILS plugin, which is capable of simultaneously displaying structures with intensities differing by several orders of magnitude. The limitations of currently available displays and visualization methods preclude viewing image data with such a dynamic range. DEVILS, implemented as a Fiji/ImageJ Plugin, homogenizes intensity differences and removes global and local background. This approach facilitates the inspection of image data with high and low intensities in a single view while at the same time allowing for the rapid inspection of volumetric 3D datasets.

The plugin modifies the pixel intensities in a non-linear way and is dependent on the size of the object, its 2D environment and the intensity itself. Intensity-based interpretation of processed image data is therefore not possible. However, it is a versatile tool for the visual inspection of large volumetric datasets, such as those typically obtained from LSFM. Prompt inspection of these datasets is key when assessing the quality of the data and deciding on further acquisition, processing and analysis strategies. Until now, a rapid method to display data with intensity differences covering several orders of magnitude was missing. DEVILS is providing an easy and straightforward approach to overcome these current limitations.

## Data availability

### Underlying data

Figshare: Data: DEVILS: a tool for the visualization of large datasets with a high dynamic range. https://doi.org/10.6084/m9.figshare.c.5197940.v2 ( Guiet *et al.*, 2020b)

This project contains the following underlying data:

- Dataset Figure Fig1, Fig3 (raw data underlying Figures 1 and 4 in czi format)
- Dataset Figure 5a, b (raw data underlying Figure 5A and 5B in czi format)
- Dataset Figure 5c (raw data underlying Figure 5C in czi format)

### Extended data

Zenodo: DEVILS: a tool for the visualization of large datasets with a high dynamic range. https://doi.org/10.5281/zenodo.4457159 ( Guiet *et al.*, 2020a).

This project contains the following extended data:

- Extended data.pdf (details of sample preparation and image acquisition for Figures 1, 4 and 5; supplementary figures)
- Workflow description.pdf (workflow describing how to process the provided “.czi” file, with BigStitcher and then with DEVILS)

Data are available under the terms of the Creative Commons Attribution 4.0 International license (CC-BY 4.0).

## Software availability

Source code available from: https://github.com/BIOP/ijp-DEVILS

Archived source code at time of publication: https://doi.org/10.5281/zenodo.4457443 ( Guiet *et al.*, 2020c).

License: GNU General Public License version 3
