# Supplementary material for: DEVILS: a tool for the visualization of large datasets with a high dynamic range
Source: F1000Res. 2021 Mar 22;9:1380. Originally published 2020 Nov 27. [Version 2] doi: 10.12688/f1000research.25447.2 (PMC8097733; doi:10.12688/f1000research.25447.2)
Supplement: Peer review history

# Peer review: Reviewer response for version 2

**DOI:** 10.5256/f1000research.54174.r82909

**Published:** 2021-05-04

**Authors:** Mehedi Masud

**Affiliations:** 1 College of Computers and Information Technology, Taif University, Taif, Saudi Arabia

- The motivation needs more clarification.
- The rationale of this tool is not clear, particularly in which domain this tool will be suitable? 
- What dataset the paper is considered?
- Is it suitable for medical datasets or what?
- The acronyms' style should be the same. See example:
  light-sheet fluorescence microscopy (LSFM)
  Display Enhancement for Visual Inspection of Large Stacks (DEVILS).

Are the conclusions about the tool and its performance adequately supported by the findings presented in the article?

Yes

Is the rationale for developing the new software tool clearly explained?

Partly

Is the description of the software tool technically sound?

Yes

Are sufficient details of the code, methods and analysis (if applicable) provided to allow replication of the software development and its use by others?

Yes

Is sufficient information provided to allow interpretation of the expected output datasets and any results generated using the tool?

Partly

Reviewer Expertise:

Deep learning for Medical diagnosis

I confirm that I have read this submission and believe that I have an appropriate level of expertise to confirm that it is of an acceptable scientific standard.

# Peer review: Reviewer response for version 2

**DOI:** 10.5256/f1000research.54174.r81839

**Published:** 2021-03-29

**Authors:** Sebastien Tosi

**Affiliations:** 1 Institute for Research in Biomedicine, IRB Barcelona, Barcelona Institute of Science and Technology (BIST), Barcelona, Spain

Thanks to the authors for addressing my comments. The only feature I would further suggest is that the plugin maintains the channel colors and composite mode settings from the original image when applied in Preview mode. Also, it would probably help to perform some automatic contrast adjustement on all channels instead of using the defaults min/max in Basic mode.

Are the conclusions about the tool and its performance adequately supported by the findings presented in the article?

Partly

Is the rationale for developing the new software tool clearly explained?

Yes

Is the description of the software tool technically sound?

Yes

Are sufficient details of the code, methods and analysis (if applicable) provided to allow replication of the software development and its use by others?

Yes

Is sufficient information provided to allow interpretation of the expected output datasets and any results generated using the tool?

Partly

Reviewer Expertise:

Signal processing, image processing, computer science

I confirm that I have read this submission and believe that I have an appropriate level of expertise to confirm that it is of an acceptable scientific standard.

# Peer review: Reviewer response for version 1

**DOI:** 10.5256/f1000research.28080.r75553

**Published:** 2020-12-17

**Authors:** Sebastien Tosi

**Affiliations:** 1 Institute for Research in Biomedicine, IRB Barcelona, Barcelona Institute of Science and Technology (BIST), Barcelona, Spain

The authors introduce an ImageJ plugin to enhance the contrast of small, dim objects in microscopy images without saturating brighter regions. Overall the technique enables a single shot view of complex images with large dynamic range without having to constantly fiddle with the contrast adjustments. The plugin is compatible with large 3D images and it somehow complements existing techniques, but its implementation suffers from several glitches that should be addressed (see Software related issues).

**Potential improvements:**

 For completeness, ImageJ Enhance Local Contrast (CLAHE) with window size adjusted to the size of largest objects should be included to the comparison (Figure 1 and related text).

 Some ImageJ native functions (e.g. subtract background) already leverage multiple cores so it is not clear what advantage brings the thread parallelization described. A speed comparison (with and without parallelization) would help. Also, the hardware used for the test report should be precisely described (especially the number of cores of the workstation).

 It is not clear why the default minimum grayscale value is set to -100. If this is due to the subtract background performed, I would recommend running it with “Disable Smoothing” and setting the default minimum grayscale value to 0.

 It is not clear what is the advantage of opening an exported folder with “Open DEVILS folder” instead of importing the image sequence. This should be motivated.

 The extra parameters of the “DEVILS Preview” dialog box should ideally only appear when ticking “Use advanced parameters” and “Largest_object_size” should not appear twice as this is rather confusing.

 I strongly suggest to better highlight the install procedure from ImageJ update sites both in the manuscript and in GitHub.

**Software related issues:**

 When running “DEVILS Preview”, the images obtained are very dim and their grayscale values do not correspond to the values actually exported when applying DEVILS with the same settings. 

 I got an error when applying the operation from DEVILS Preview: “Unrecognized command “DEVILS Preview”.

**Wording:**

 The words used throughout the text should be more consistent, for instance: “DEVILS Workflow”, “DEVILS Routine”, “DEVILS Algorithm”, “DEVILS operation” or “Slice”, “Plane”, “Z-Plane” should be unified.

 This sentence should be rephrased: “A popular method to remove intensity values from large objects is to divide the image by its blurred version”.

Are the conclusions about the tool and its performance adequately supported by the findings presented in the article?

Partly

Is the rationale for developing the new software tool clearly explained?

Yes

Is the description of the software tool technically sound?

Yes

Are sufficient details of the code, methods and analysis (if applicable) provided to allow replication of the software development and its use by others?

Yes

Is sufficient information provided to allow interpretation of the expected output datasets and any results generated using the tool?

Partly

Reviewer Expertise:

Signal processing, image processing, computer science

I confirm that I have read this submission and believe that I have an appropriate level of expertise to confirm that it is of an acceptable scientific standard, however I have significant reservations, as outlined above.

# Response

**Published:** 2021-01-22

**Authors:** Arne Seitz

Dear Sebastien,

 Thanks for carefully reading our manuscript and your useful comments and suggestions. We are grateful for them and convinced that they help to improve the quality of the publication as well as its understandability as well as the DEVILS plugin.

 Whenever possible we addressed your points either in the new version of the manuscript or the supplemental material. Please find the answers to the individual points you mentioned in the following:

**1. For completeness, ImageJ Enhance Local Contrast (CLAHE) with window size adjusted to the size of largest objects should be included to the comparison (Figure 1 and related text).**

 We have added a comparison with ImageJ enhance Local Contrast (CLAHE) in the supplemental material and are referring to it in the main text.

 We found that this approach can give equally good results however that the parameter finetuning can be time-consuming. Therefore, we think that DEVILS has some advantages for the visualization of large volumetric datasets.

**2. Some ImageJ native functions (e.g. subtract background) already leverage multiple cores so it is not clear what advantage brings the thread parallelization described. A speed comparison (with and without parallelization) would help. Also, the hardware used for the test report should be precisely described (especially the number of cores of the workstation).**

 DEVILS is written so that multiple image planes can be processed in parallel in order to speed up the workflow.

 We have investigated the effect of further parallelization and added the results to the supplemental material section. 

**3. It is not clear why the default minimum grayscale value is set to -100. If this is due to the subtract background performed, I would recommend running it with “Disable Smoothing” and setting the default minimum grayscale value to 0.**

 We have taken up the suggestion and evaluated the “Disable Smoothing” option. It modifies the resulting image, however negative values are still present as shown by the macro accessible via this gist link, the macro can also be run with the gist link in ij.imjoy.io

**4. It is not clear what is the advantage of opening an exported folder with “Open DEVILS folder” instead of importing the image sequence. This should be motivated.**

 The advantage of the “Open DEVILS folder” is to be able to import processed images as stack or hyperstack directly without the need to specify the order (channels, time, slices). This is in particular useful for multidimensional datasets. We have updated the documentation to explain the advantages of using this folder instead of importing the images manually.

https://github.com/BIOP/ijp-DEVILS#opening-tiff-files--opening-devils-folder

**5. The extra parameters of the “DEVILS Preview” dialog box should ideally only appear when ticking “Use advanced parameters” and “Largest_object_size” should not appear twice as this is rather confusing.**

 We created two separate commands: DEVILS Preview (Basic) and DEVILS Preview (Advanced) in order to avoid appearing the parameter twice. This helps to avoid the above mentioned confusion.

**6. I strongly suggest to better highlight the install procedure from ImageJ update sites both in the manuscript and in GitHub.**

 The documentation is now containing a paragraph about the installation procedure. ( https://github.com/BIOP/ijp-DEVILS#installation ).

**7. When running “DEVILS Preview”, the images obtained are very dim and their grayscale values do not correspond to the values actually exported when applying DEVILS with the same settings.**

 The preview is interactive, and the “Brightness and Contrast” menu can be used to adjust the min and max display range for each channel. This has been clarified in the documentation.

https://github.com/BIOP/ijp-DEVILS#devils-preview--advanced-and https://github.com/BIOP/ijp-DEVILS#minimum-resp-maximum-for-final-conversion-step 

**8. I got an error when applying the operation from DEVILS Preview: “Unrecognized command “DEVILS Preview”.**

 This bug his has been fixed in the latest version available via the update site. 

**9. The words used throughout the text should be more consistent, for instance: “DEVILS Workflow”, “DEVILS Routine”, “DEVILS Algorithm”, “DEVILS operation” or “Slice”, “Plane”, “Z-Plane” should be unified.**

 The manuscript was changed by using the term “DEVILS plugin” and avoiding the other ones. 

**10. This sentence should be rephrased: “A popular method to remove intensity values from large objects is to divide the image by its blurred version”.**

 The sentence was rephrased.

 Thanks again for your comments and suggestions.

 Best regards
